# Toxicological Risks of Agrochemical Spray Adjuvants: Organosilicone Surfactants May Not Be Safe

**DOI:** 10.3389/fpubh.2016.00092

**Published:** 2016-05-11

**Authors:** Christopher A. Mullin, Julia D. Fine, Ryan D. Reynolds, Maryann T. Frazier

**Affiliations:** ^1^Department of Entomology, Center for Pollinator Research, The Pennsylvania State University, University Park, PA, USA

**Keywords:** adjuvant, agrochemical formulation, organosilicone surfactant, non-target effects, spray tank mix

## Abstract

Agrochemical risk assessment that takes into account only pesticide active ingredients without the spray adjuvants commonly used in their application will miss important toxicity outcomes detrimental to non-target species, including humans. Lack of disclosure of adjuvant and formulation ingredients coupled with a lack of adequate analytical methods constrains the assessment of total chemical load on beneficial organisms and the environment. Adjuvants generally enhance the pesticidal efficacy and inadvertently the non-target effects of the active ingredient. Spray adjuvants are largely assumed to be biologically inert and are not registered by the USA EPA, leaving their regulation and monitoring to individual states. Organosilicone surfactants are the most potent adjuvants and super-penetrants available to growers. Based on the data for agrochemical applications to almonds from California Department of Pesticide Regulation, there has been increasing use of adjuvants, particularly organosilicone surfactants, during bloom when two-thirds of USA honey bee colonies are present. Increased tank mixing of these with ergosterol biosynthesis inhibitors and other fungicides and with insect growth regulator insecticides may be associated with recent USA honey bee declines. This database archives every application of a spray tank adjuvant with detail that is unprecedented globally. Organosilicone surfactants are good stand alone pesticides, toxic to bees, and are also present in drug and personal care products, particularly shampoos, and thus represent an important component of the chemical landscape to which pollinators and humans are exposed. This mini review is the first to possibly link spray adjuvant use with declining health of honey bee populations.

## Introduction

Applications of modern pesticide formulations, particularly in combinations, are often accomplished using proprietary spray adjuvants to achieve high efficacy for targeted pests and diseases (1). An adjuvant is an additive or supplement used to enhance the performance or aid in the stability of formulations of active ingredients (2). Adjuvant products are formulated combinations of surfactants, penetrant enhancers, activators, spreaders, stickers, cosolvents, wetting agents, pH modifiers, defoaming agents, drift retardants, nutrients, etc., depending on their proposed utility. Usually, adjuvants are much less expensive than formulated active ingredients and can reduce the active ingredient dose needed by an order or more of magnitude (3, 4). Similarly, contemporary drug delivery to humans and animals transdermally (5) and orally (6) is often mediated *via* adjuvant technologies that enhance penetration. Newer agrochemical technologies include co-formulants such as polyethoxylated tallow amines, cosolvents such as *N*-methyl-2-pyrrolidone (NMP), and spray adjuvants such as organosilicone polyethoxylates (7).

Numerous studies have found that pesticide active ingredients elicit very different physiological effects on non-target organisms when combined with their co-formulants and tank adjuvants (7–9). Despite the widespread assumption that formulation ingredients and spray adjuvants are biologically inert, substantial evidence suggests that this is often not the case. Indeed, glyphosate has weak ecotoxicity and systemic movement without tallow amines and other adjuvants (10–12), including its toxicity to mammals (13) and human cells (14). Noteworthy is the fact that spray tank adjuvants by themselves harm non-target organisms from all taxa studied. Adjuvant-dependent toxicities, often more than the associated formulations of herbicides and fungicides, have been reported for bacteria (15), cyanobacteria (16), algae (17), and snails (18). The non-ionic spray adjuvant R-11 synergized the acute toxicity of the insecticides spinosad (19) and imidacloprid (20) on aquatic crustaceans and, in the absence of an insecticide, reduced the growth rate of *Daphnia pulex* at relevant field concentrations found after application near aquatic systems (21). Aquatic organisms are particularly vulnerable to the general ecotoxicity of adjuvant surfactants ranging from invertebrates (22, 23) to fish (19, 24, 25) and amphibians (26). Terrestrial insects, in turn, have long been shown susceptible to insecticide synergisms associated with spray adjuvants (27, 28). Many of the classical cases of ecotoxicities found with spray adjuvants and used with pesticides other than glyphosate are due to older surfactant classes, such as nonylphenol polyethoxylates, which environmentally degrade to the endocrine disrupting nonylphenols (29). It is clear that agrochemical risk assessment that takes into account only pesticide active ingredients and their formulations in absence of the spray adjuvants commonly used in their application (30, 31) will miss important toxicity outcomes that may prove detrimental, even to humans. Here, we attempt to characterize the scope of spray adjuvant use, especially organosilicone surfactants, and explore a possible link between their increasing presence in California almonds and the declining health of honey bee populations.

## Spray Adjuvants Contribute to the Toxic Load

Supplemental adjuvants used in tank mixes generally enhance the pesticidal efficacy as well as inadvertently the non-target effects of the active ingredient after application (7, 14). Dramatic impacts of agrochemical formulants on the bee toxicity of pesticide active ingredients have been documented (32). Formulations are generally more toxic than active ingredients, particularly fungicides, by up to 26,000-fold based on published literature. The highest oral toxicity of three insecticide formulations tested was for Vertimec^®^ 18 EC that was 8,970 times more toxic to the stingless bee *Melipona quadrifasciata* and 709 times more toxic to the honey bee than the topically applied active ingredient abamectin in acetone (33). However, the largest documented formulation compared to active ingredient differences in bee toxicity have been with the least toxic pesticides, particularly fungicides. Among the 300 pesticide formulations tested for oral toxicity to adult honey bee in China, a 25% EC formulation of the fungicide tebuconazole was equally toxic to the most bee-toxic insecticide known, emamectin benzoate (LD_50_ = 0.0035 μg/bee), whereas a 5% suspension concentrate of tebuconazole was > 25,000 times less toxic (34). This product-dependent range in toxicity is presumably determined by the undisclosed fungicide co-formulants. While technical glyphosate has virtually no toxicity for honey bees, common formulations such as WeatherMAX^®^ do (35). Commercial formulations of fumagillin acid used to control *Nosema* and other microsporidian fungal diseases in honey bees and mammals, respectively, are actually salts of the base dicyclohexylamine. This co-formulant is five times more toxic and persistent than the active ingredient to rodents and other organisms, serving as a sensitive bioindicator of fumagillin pollution (36). Most studies documenting pesticide effects on honey bees are performed without the formulation or other relevant spray adjuvant components used when applying the active ingredient, most often due to lack of such required tests for product registration (7).

Less potent bee toxicities are usually found when spray adjuvants are tested alone or relative to the pesticide formulations used in tank combinations. About one-third of non-ionic, organosilicone and other surfactant spray adjuvants at up to a 0.2% aqueous solution have been shown to deter or kill honey bees (37–39). Exposure to the nonylphenol polyethoxylate adjuvant N-90 by itself at field rates impaired nest recognition behavior of two managed solitary bees, *Osmia lignaria* and *Megachile rotundata* (40). While the organosilicone adjuvant Break-Thru^®^ fed to nurse bees at 200 ppm with or without 400 ppm of the fungicide Pristine^®^ did not impact honey bee queen development or survival (41), toxic interaction of the co-occurring insect growth regulator (IGR) dimilin with this adjuvant is likely [cf., Ref. (42)]. Higher toxicities were found when honey bees are fed related commercial organosilicone surfactants in 50% sucrose with oral LC_50_s around 10 ppm (7). A discontinued agrochemical surfactant perfluorooctylsulfonic acid is highly and orally toxic to *Bombus terrestris* (43). The penetration enhancing solvent NMP commonly present in agrochemical formulations is a dietary toxicant for honey bee larvae at 100 ppm (44).

Organosilicone surfactants are particularly potent as super-penetrants, super-spreaders, and probable ecotoxicants (7). They are used worldwide at up to 1% (10,000 ppm) of the spray tank mix, while other adjuvant classes require higher amounts up to 5% of the spray tank mix (3, 32). All organosilicone surfactant adjuvants (OSSA) tested (Dyne-Amic^®^, Syl-Tac^®^, Sylgard 309^®^, and Silwet L-77^®^) impaired honey bee olfactory learning much more than other non-ionic adjuvants (Activator 90^®^, R-11^®^, and Induce^®^), while the crop oil concentrates (Penetrator^®^, Agri-Dex^®^, and Crop Oil Concentrate^®^) were inactive at 20 μg per bee (45). The greater surfactancy of organosilicones over other non-ionic adjuvants and crop oil concentrates can drive the stomatal uptake of large bacterial-sized mineral particles (46) and *Agrobacterium* transformation of grape plantlets (47), and thus may aid movement of pathogens into bee tissues.

## Spray Adjuvant Use during Pollination of California Almonds

Pollination of California almonds during February and March is the single largest pollination event in the world. Over 60% (1.5 million) of USA honey bee colonies are transported to California each year to pollinate the crop. A workshop convened to address reduced overwinter survivorship of commercial honey bee colonies used in almond pollination since the 2006 onset of colony collapse disorder (CCD) judged neonicotinoids unlikely to be a sole factor and *Varroa* mites plus viruses to be a probable cause (48). However, fungicides, herbicides, and spray adjuvants were not evaluated. Recent surveys of migratory beekeepers who pollinate almonds do not self-report overwintering losses greater than the majority of non-migratory beekeepers, although their summer colony losses tend to be higher (49). Better management practices employed by migratory beekeepers who pollinate almonds may explain their lower winter losses in comparison with sideline or backyard beekeepers (50). Nevertheless, it has been surmised by beekeepers and documented by researchers that decreasing honey bee health issues are initiated in almonds, a winter/early spring pollinated crop, and then progressed over the course of the year as colonies are employed to pollinate other crops including apples, blueberries, alfalfa, cotton, pumpkin, cantaloupe, etc. Although the rates of foraging honey bees were not reduced over time during almond pollination in contrast to those pollinating cotton and alfalfa, there was no corresponding increase in foraging population though a significant increase in colony size occurred (51). Some of the highest pesticide residues, especially fungicides, were found on almonds, which represents a notable pesticide exposure risk and ranked fifth in hazard among the eight crops assessed (51). Ironically, increasing fungicide load in pollen has been associated with increased probability of fungal *Nosema* infection in exposed bees (52).

California law defines adjuvants packaged and sold separately as pesticide products that require registration (53). Every application of a spray tank adjuvant is reported with detail that is unprecedented globally. California almond exposes most USA honey bees to highly documented pesticide and adjuvant applications and is an unique crop to assess all other agrochemical inputs in the absence of neonicotinoids, presently considered to be the primary pesticide factor associated with pollinator decline (54). There are no substantial applications of neonicotinoids to this monoculture (55), particularly when honey bees are present, and almond pollen and nectar tend to be the sole food source unless supplemental sugar feeding is employed (52). Pesticide usage information for California has been archived since 1990 in the pesticide use reporting (PUR) database maintained by the California Department of Pesticide Regulations (55). The great utility of this data for assessing environmental risks of spatial and temporal pesticide use in California almonds to aquatic organisms and earthworms has been demonstrated (56). However, our study is the first to include spray adjuvants as potentially toxic agrochemical inputs in risk evaluation.

**Figure 1:**
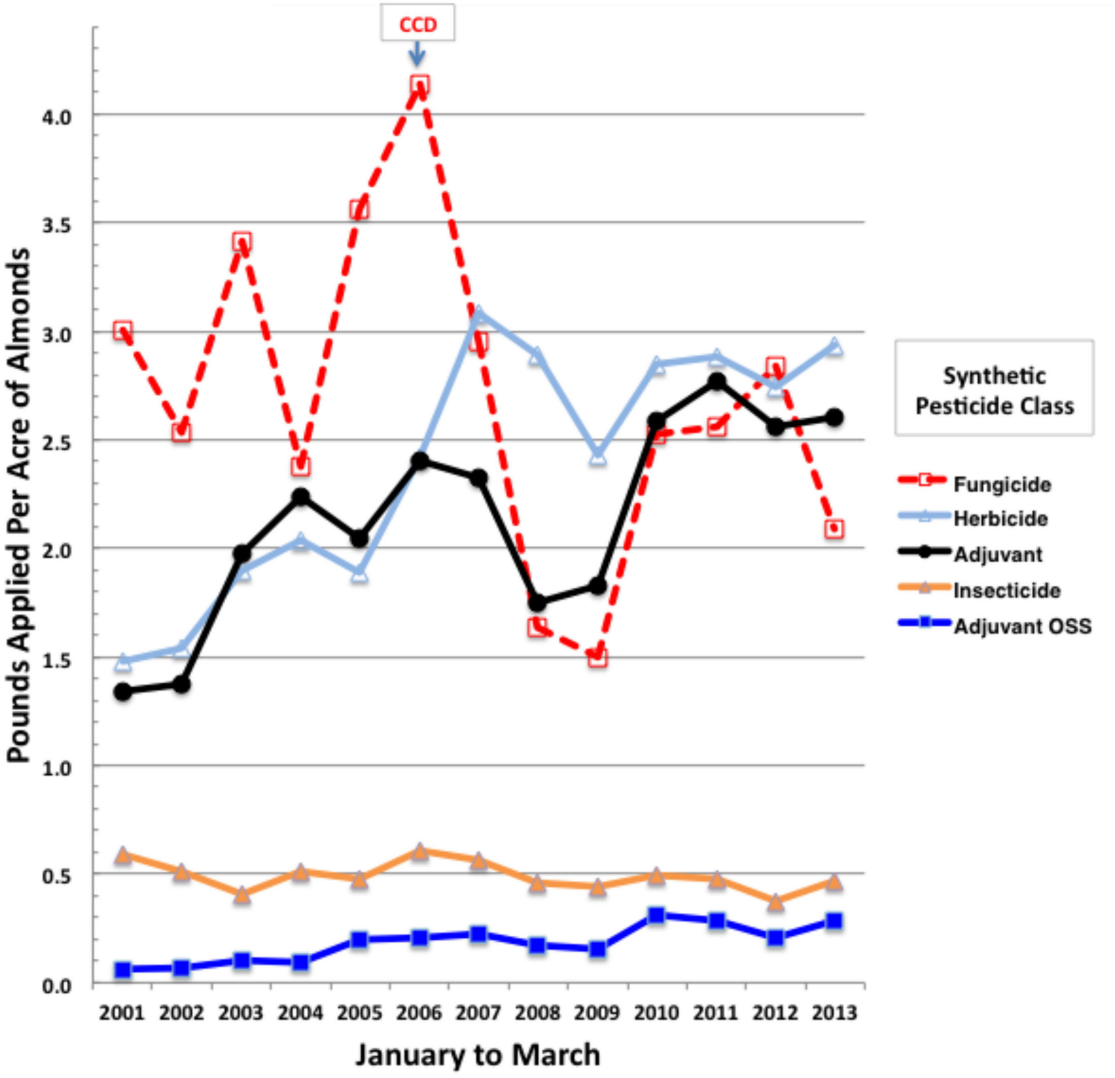
**Total pounds of synthetic pesticides by class applied per acre of California almonds during January to March of 2001 through 2013**. Yearly total almond bearing acres were from the CA Department of Food and Agriculture (57).

We analyzed annual trends in applications of tank adjuvants and associated formulated products of active ingredients during almond pollination (February and March). January applications were also included since their foliar residues may pose toxicity risks for newly arriving bee colonies. Over 3.3 million records for almond applications were downloaded from PUR (55) and sorted for January to March of 2001–2013 using Microsoft Excel (Mac 2011). Only synthetic pesticides were analyzed for trends, thereby excluding bulky applications of older natural products and biologicals, such as sulfur, petroleum and mineral oils, copper salts, and microbials, since CCD was first noted in 2006, decades after major regular inputs of these natural pesticides were initiated. While overall statewide synthetic fungicide and insecticide use on almonds has not increased over this evaluation period, applications of herbicides and spray adjuvants, the latter including nutrient and buffer supplements, have doubled (Figure 1). Yearly application rates were normalized to total almond bearing acres, which increased from 530,000 in 2001 to 850,000 acres in 2013 (57), indicating that the total synthetic pesticide load has increased on almonds since the onset of CCD (Figure 1). Because herbicide applications are generally made to the understory and not to the flowering canopy where pollinator exposure is likely, we focused on actual tank adjuvant and pesticide mixes that may provide direct exposure risks for bees. Among adjuvant classes, the organosilicone surfactants pose the greatest toxicity risks for honey bees (7).

**Figure 2:**
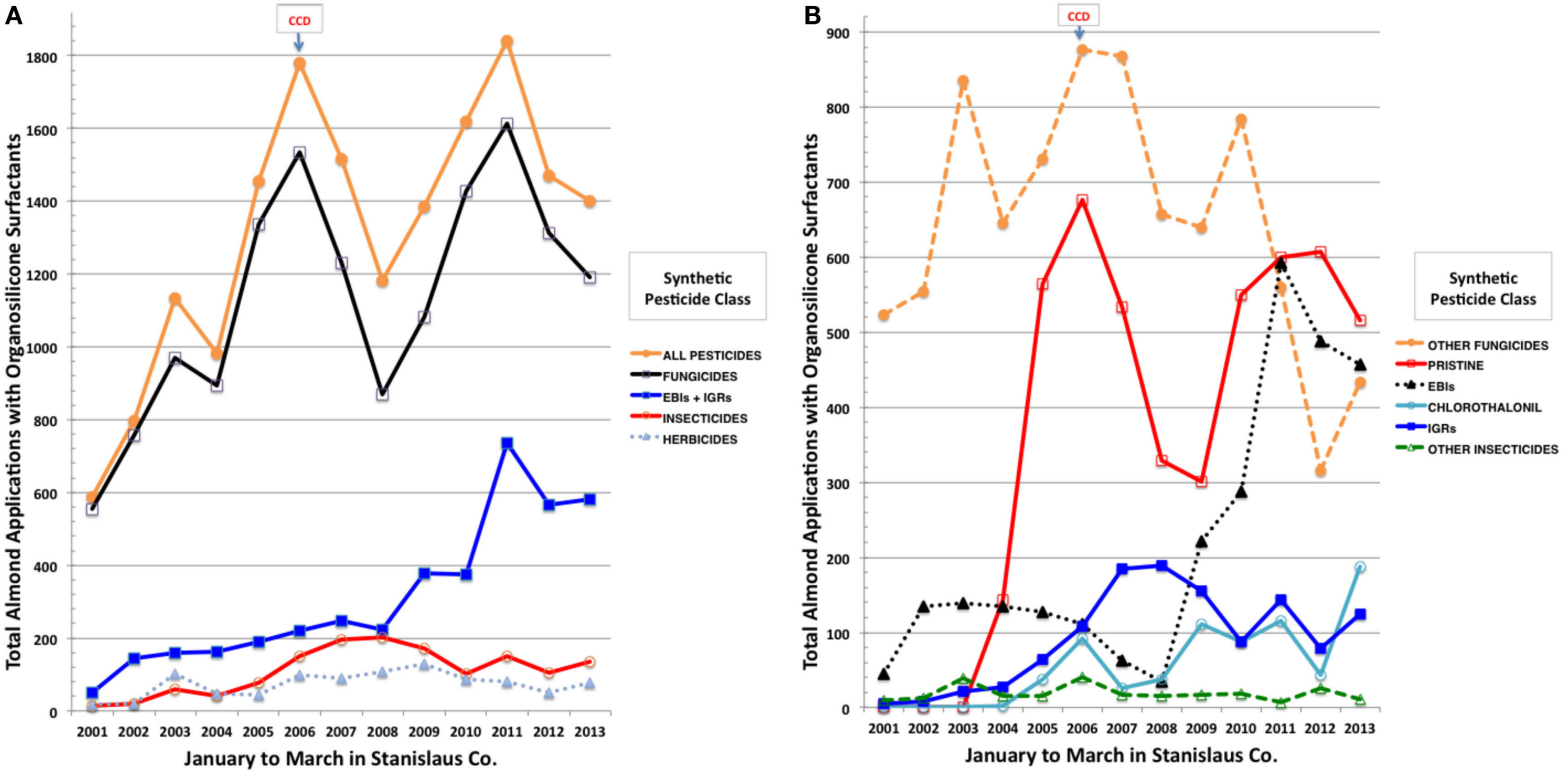
**Total applications to almonds in Stanislaus County, CA, USA of synthetic pesticides by class (A) and by more selective class and individual pesticides (B) during January to March of 2001 through 2013 using an organsilicone surfactant**.

We then conducted a detailed analysis of temporal trends in organosilicone applications for Stanislaus Co., a major almond producing county in California (57), which had the largest number of pesticide applications over our evaluation period. PUR records (55) were sorted by date, county/meridian/township/range/section (COMTRS) location, and amount of treated almond acres. Co-occurring and synonymous records were assumed to represent combined pesticide and adjuvant products within the same tank application mix. Based on this premise, most of the spray combinations comprised, in addition to one or more pesticide formulations, at least one tank adjuvant. Focused assessment was then made out of the total number and percentages of applications containing an OSSA, which included 45 products (Table S1 in Supplementary Material) dominated by Dyne-Amic^®^, Syl-Tac^®^, Sylgard 309^®^, RNA Si 100^®^, First Choice Break-Thru^®^, Freeway^®^, Kinetic^®^, Multi-Spred^®^, Widespread Max^®^, and Silwet L-77^®^. Similar combinations of products were assigned unique tank mix codes and resorted. Almost 10,000 pesticide applications on almonds in Stanislaus Co. contained an OSSA over the years evaluated, each on average to 40 acres. The greatest increase in major agrochemical inputs observed before and after onset of CCD in 2006 was the tripling of total pesticide applications containing an OSSA from 587 in January–March 2001 to 1,781 in January–March 2006 (Figure 2A). Greater than 80% of these applications contained fungicides, followed by 10% insecticides, and 5% herbicides. Ergosterol biosynthesis inhibitor (EBI) fungicides and IGR insecticides were greatly increased, whereas herbicide and other insecticide applications were fairly static across this period (Figures 2A,B). Pristine^®^ (a combination of boscalid and pyraclostrobin), chlorothalonil, and EBIs (propiconazole > myclobutanil > fenbuconazole > metconazole > difenoconazole) dominated the increasing trends in fungicide use at the onset of CCD (Figure 2B). The IGRs (diflubenzuron > methoxyfenozide > pyriproxyfen > tebufenozide) displayed the greatest increases among insecticides in spray tank mixes containing OSSA during the onset and continuation of CCD (Figure 2B). Concomitantly, greatest decreasing tendencies in almond pesticide applications were for other fungicides (cyprodinil, iprodione, and azoxystrobin) and the older EBI myclobutanil, while inputs of herbicides (primarily glyphosate, oxyfluorfen, and paraquat) with OSSA did not change markedly. Based on the CDPR data for agrochemical applications to California almonds during pollination, increasing adjuvant use, particularly the OSSAs, in tank mixes with fungicides, including EBIs, Pristine^®^, and chlorothalonil, and with IGR insecticides may be associated with recent USA honey bee declines.

## Organosilicones: The Most Powerful Surfactants

Organosilicone surfactants are the most potent adjuvants and super-penetrants available to growers (58, 59). These polyethoxylates and those containing the nonyl- and octylphenols are widely used as non-ionic surfactants in spray adjuvants or additives in agrochemical formulations applied during bloom when bees are foraging. Organosiloxane surfactants were detected in all wax samples and 60% of pollen samples, although absent from honey (60). Their general wide occurrence as residues in beehive samples is noteworthy since spray adjuvants are not presently regulated by the EPA (61). Nonylphenol more than organosiloxane and octylphenol polyethoxylates were found in wax samples, while pollen and particularly honey residues were lower (62). Major commercial spray tank adjuvants are blends of organosilicone, nonylphenol, and octylphenol polyethoxylates, making it more difficult to associate environment residues with any specific product (63). Nevertheless, sample levels of the more abundant nonylphenol polyethoxylate residues may be used as a risk predictor for pesticide exposure because of their frequent coincidence in tank mixes of formulations and adjuvants (62). Spray tank adjuvants containing these polyethoxylates greatly influence pesticide fate (64) in pollinator or other environments, generally increasing the residue levels, of particularly fungicides (65) and herbicides (66), available to expose pollinators and other non-target species. The impact of OSSA sprays on the frequent incidence of neonicotinoid residues in bee environments (67) and their often associated roles in pollinator decline (68) may be great since the highest imidacloprid residue ever reported in pollen (7.4 ppm) was after use of Dyne-Amic^®^ on citrus [(69), Appendix E].

Even at 10 ppm, OSSAs are good, stand-alone insecticides and miticides (7, 70), and can be more toxic to beneficial insects than the active ingredient used to control the associated pest (71). Silwet L-77^®^ and Kinetic^®^ are known to synergize the neonicotinoid imidacloprid used to control the psyllid vector of citrus greening disease (72). Yearly use of these potent adjuvants continues to increase, with an estimated annual global production of 1.3 billion pounds of OSSAs in 2008 among 10 billion pounds of all organosilicones (73). This is 30 times greater than the highest estimated global annual imidacloprid use of 44 million pounds (74). Silwet L-77^®^ was the most potent endocrine disruptor among surfactants tested in a screen of 1,814 chemicals, with composite scores that placed it in the top 38 of the 465 endocrine disruptors found [(75), supplemental data], much more active than polyoxyethylene(10)nonylphenyl ether. All six neonicotinoids, including imidacloprid, were inactive in the entire battery of endocrine tests used. Organosilicone surfactants are also present in drug and personal care products, particularly shampoos (76), and thus represent an important component of the chemical landscape to which bees (32) and humans (77) are exposed. These widely used super surfactants readily move across membranes, become systemic in plants and animals, and can ultimately degrade to silica (78) causing silicosis in sensitive tissues of exposed organisms.

## Are Organosilicone Surfactants Causing Harm and Underregulated?

Organosilicone surfactants are the “gold” standard for effecting solution of complex mixtures of agrochemical components of wide-ranging polarites in the spray tank. Hundreds of thousands of pounds of organosilicone adjuvants are applied every year on almonds in California alone (7, 45), both during and subsequent to bloom when bee pollinators are present. The high incidence of OSSAs in USA beehives and their ability to impair adult learning and be toxic to honey bees at all stages of development points to their great potential to harm bees and other non-target species, and yet, they are typically not even considered in the risk assessment process. It is clear that relevant pesticide risk assessment for pollinators and other non-target species cannot be addressed solely by evaluating the active ingredients without the concomitant formulation ingredients and spray tank adjuvants. Lack of risk mitigation on spray tank adjuvants presently allows major OSSA products such as Break-Thru^®^, Kinetic^®^, RNA Si 100^®^, Silwet Eco Spreader^®^, Syl-Coat^®^, and Widespread^®^ to be used on any “organic” crop under a certified Organic Materials Review Institute (OMRI) label (79).

Spray adjuvants are largely assumed to be biologically inert and are not registered by EPA at the federal level in the USA (7, 55). Registration and monitoring of adjuvant use patterns are regulated at the state level in the USA, and most states do not participate in this process. To the best of our knowledge, only California, Washington, and perhaps Oregon make substantial effort to monitor use patterns or regulate these major chemical inputs into the environmental landscape. This lack of federal oversight is surprising since Department of Transportation employees of Pennsylvania and Iowa claim that herbicide applications to right-of-ways and roadways always contain a separate spray tank adjuvant (personal communications, 2015). Leaving regulation to the mandate of individual states results in a “wild west” approach that, in most cases, leaves these chemicals unaccounted for and allows for their increasing presence in our environment. Requiring regulation of spray tank adjuvants at the federal level in the USA would be a reasonable step toward addressing this problem.

While we recognize that chemical stressors alone are likely not responsible for the decline of pollinator or other non-target organisms, the true impact of chemical exposure is impossible to determine given our lack of understanding of the total chemical burden, a burden that clearly includes unknown and unevaluated materials. Coincidence of virus and pesticide exposures in declining honey bee colonies (80) is most noteworthy among other factors, which also includes malnutrition and elevated *Varroa* mites. More industry and regulatory agency disclosure of the identity of agrochemical adjuvant and formulation components would aid in evaluating risk and hazard assessment. Most adjuvants and inert ingredients are presently exempted from human safety tolerances, generally recognized as safe, and thus no environmental monitoring is required (7). A needed improvement is to include all formulation (81) and adjuvant (82) ingredients at relevant environmental input and exposure levels, and not just active ingredients, in studies to document the safety and risk for pollinators and other non-target species prior to product registration and commercialization.

## Author Contributions

CM and MF were the primary authors and contributed substantially to the concept, design, final drafting, and primary accountability of the content of this mini review. JF and RR were key to the acquisition, analysis, and interpretation of cited data and were involved in drafting and final approval for work cited here.

## Conflict of Interest Statement

The authors declare that the research was conducted in the absence of any commercial or financial relationships that could be construed as a potential conflict of interest.

## Abbreviations

AI, active ingredient; CCD, colony collapse disorder; EBI, ergosterol biosynthesis inhibitor; EC, emulsifiable concentrate; IGR, insect growth regulator; NMP, *N*-methyl-2-pyrrolidone; OSSA, organosilicone surfactant adjuvant; ppm, part per million.
