# Supplementary material for: Toxicological Risks of Agrochemical Spray Adjuvants: Organosilicone Surfactants May Not Be Safe
Source: Front Public Health. 2016 May 11;4:92. doi: 10.3389/fpubh.2016.00092 (PMC4862968; doi:10.3389/fpubh.2016.00092)
Supplement: Supplementary file 1 [file table_1.docx]

**Table S1 |** **List of organosilicone surfactant products used on**

**California almonds during January to March of 2001 through 2013.**

#233 Wet-Sol Concentrate^®^
Aero Dyne-Amic^®^
Ag Rx Multi-Spred^®^
Bac Spred Stik^®^
Break-Thru^®^
Britz Fenish Silicone Surfactant^®^
Britz Silglow^®^
Broad Spred^®^
Bronc Plus Dry-Edt^®^
Cadence^®^
CMR Can-Hance^®^
CMR Silicone Surfactant^®^
Dyne-Amic^®^
Faststrike^®^
First Choice Break-Thru^®^
First Choice Hi-Wett^®^
First Choice Solar Methylated Seed Oil With Organosilicone^®^
Foam Fighter^®^
Freeway^®^
Hi-Wett Super-Spreader^®^
Kinetic^®^
Metho-Sil^®^
Multi-Spred^®^
Organosilicone MVO Modified Vegetable Oil And Surfactant Blend^®^
Phase^®^
PHT Faststrike^®^
PHT Quark^®^
PHT Silglow^®^
Pro Multi-Spred^®^
Pro Silicone 100^®^
Quark^®^
RNA Si 100^®^
Silicone Super Wetter^®^
Silicone Surfactant^®^
Silkin^®^
Silwet 806^®^
Silwet L-77^®^
Syl-Coat^®^
Syl-Tac^®^
Sylgard 309^®^
Tactic^®^
Unfoamer^®^
Vader^®^
Widespread^®^
Widespread Max^®^

Based on Pesticide Use Reporting database of California Department of Pesticide Regulations (55).
